# Doctor-patient interaction in Finnish primary health care as perceived by first year medical students

**DOI:** 10.1186/1472-6920-5-34

**Published:** 2005-09-15

**Authors:** Juhani Miettola, Pekka Mäntyselkä, Tuula Vaskilampi

**Affiliations:** 1 Department of Public Health and General Practice, University of Kuopio, P.O.Box 1627, 70211 Kuopio, Finland; 2 Unit of General Practice, Kuopio University Hospital, P.O.Box 1777, 70211 Kuopio, Finland

## Abstract

**Background:**

In Finland, public health care is the responsibility of primary health care centres, which render a wide range of community level preventive, curative and rehabilitative medical care. Since 1990's, medical studies have involved early familiarization of medical students with general practice from the beginning of the studies, as this pre-clinical familiarisation helps medical students understand patients as human beings, recognise the importance of the doctor-patient relationship and identify practicing general practitioners (GPs) as role models for their professional development. Focused on doctor-patient relationship, we analysed the reports of 2002 first year medical students in the University of Kuopio. The students observed GPs' work during their 2-day visit to primary health care centres.

**Methods:**

We analysed systematically the texts of 127 written reports of 2002, which represents 95.5% of the 133 first year pre-clinical medical students reports. The reports of 2003 (N = 118) and 2004 (N = 130) were used as reference material.

**Results:**

Majority of the students reported GPs as positive role models. Some students reported GPs' poor attitudes, which they, however, regarded as a learning opportunity. Students generally observed a great variety of responsibilities in general practice, and expressed admiration for the skills and abilities required. They appreciated the GPs' interest in patients concerns. GPs' communication styles were found to vary considerably. Students reported some factors disturbing the consultation session, such as the GP staring at the computer screen and other team members entering the room. Working with marginalized groups, the chronically and terminally ill, and dying patients was seen as an area for development in the busy Finnish primary health care centres.

**Conclusion:**

During the analysis, we discovered that medical students' perceptions in this study are in line with the previous findings about the importance of role model (good or bad) in making good doctors. Therefore, medical students' pre-clinical primary health care centre visits may influence their attitudes towards primary health care work and the doctor-patient relationship. We welcome more European studies on the role of early pre-clinical general practice exposure on medical students' primary care specialty choice.

## Background

In Finland, public health care is the responsibility of primary health care centres which render a wide range of first level preventive, curative and rehabilitative medical care. In order to improve continuity of care, most Finnish municipalities have switched from the traditional primary health-care system to a family doctor system [1,2]. In Finland, medical studies have traditionally involved an initial two-year preclinical period of mainly theoretical courses. However, students now have contact with patients from the beginning of their studies [3].

The first international reports on the subject of doctor-patient relationship training during pre-clinical medical education originate from the 1970s [4]. Presently, medical schools in several countries integrate training in communication skills into the medical curriculum, and provide medical students with the opportunity to meet patients in a real primary health care setting during the early stages of their studies [5,6]. Pre-clinical familiarisation of medical students with general practice considerably improves students' communication skills and understanding of patients' perceptions, helps students understand patients as human beings, and helps them recognise the importance of the doctor-patient relationship [7-9]. There is also much international documentation concerning the importance of role models in making good doctors [10]. However, some international reports claim that early exposure of medical students to family practice faculty or to family practitioners in their own clinics does not influence primary care specialty choice [11].

In Finnish society, there are quite alarming signs of a fall in public appreciation of public primary health care [12]. Authorities have devoted more attention to efforts to restore the traditional high esteem in which the demanding work of general practitioners and their teams in primary health care centres has been held. Medical schools have also acknowledged the problem.

As a continuation of the community oriented medical training program since early 70's at the University of Kuopio, a two-day primary health care centre visit was introduced in 1996 to the *Introduction to Medicine Course*, which is held in the first year of the medical studies. The purpose of the field visit is to familiarise students with the Finnish health care system and its operation, the everyday work of GPs, and in particular what they do and how they do it in their interaction with patients. Moreover, the purpose is to underline the significance of the interaction between doctor and patient. The orientation programme involves theoretical and practical teaching of communication skills prior to the primary health care centre visit.

In the primary health care centres, students observe the operation of the institution and the work of GPs alone and as a team member, interview one primary care patient and write a report about their findings based on the accompanying checklist (Table 1). In the reports, students also make their comments on the doctor-patient interaction. They observe encounter situations and report their perceptions based on the checklist.

**Table 1:** Check-list for the health care centre visit (the focus areas of this study with bold font).

Location of the health care centre
Catchment area and population
Number of general and other medical practitioners
Contents of the working day of a general practitioner
working in different sectors (accident and emergency department, outpatient department, maternal, child, school, occupational health care, family planning, home care, wards)
participation in training, meetings and workshops
Attitude of the practitioners towards their work
**Attitude of the practitioners towards their patients**
**Doctor-patient communication**
Practitioner as a team member
teamwork
attitude towards colleagues, nurses, physiotherapists and other key persons
Students' personal experiences of the visit

In our study, we focused on 1^st ^year medical students' perceptions of the attitude of the general practitioners towards their patients and doctor-patient communication. We aimed at reaching students' own experiences, perceptions and meanings as original as possible. Therefore, we gave them only a tentative list of the main themes (categories) to be observed and emphasised their freedom to express themselves openly. In our study, there was no theoretical guiding principle, but we utilised Garfinkel's (1967) ethnometodological ideas as the source on gradually development of theoretical thinking [13].

## Methods

In 2002, 2003 and 2004 like in the previous years since 1996, students observed the work of GPs in eastern and central Finland. As a part of their assignment, they wrote a short report of their experiences.

In 2002, altogether 64 primary health care centres were involved in the programme, of which 35 are official university partner institutions. Of the 133 first year students, 127 students (95.5 % of all in the class) submitted their reports, of which 88 were female students (69 %) and 39 male students (31 %). In the reference years 2003 and 2004, we received reports from 118 students (92.2% of 128 students) and 130 students (98.5% of 132 students) respectively.

Each author (JM, PM, TV) worked out one third of the 2002 reports. The texts were analysed independently and systematically by each author finding out the main themes/categories. All authors found out the same main categories, namely doctor-patient relationship, doctor as an ideal role model, perception on their own medical capabilities, observations and on organisational setting of the primary health care centre. Finally, the contents were coded by agreed subcategories (Table 2).

**Table 2:** The categories of doctor-patient interaction.

A. Personal qualities of practitioners
general interest, empathy, adaptability, approach
personality factors
B. Communication, interaction, dialogue
communication styles
patient information, authority
influence of continuity of care in doctor-patient relationship
disturbances in doctor-patient communication
C. Context-related topics
specific patient groups
chronic and terminal illness, dying patients
physical and psychological environment
haste
teamwork/working alone
comprehensiveness of the work
organisational issues
list system versus conventional system
continuity of care

**Figure 1:**
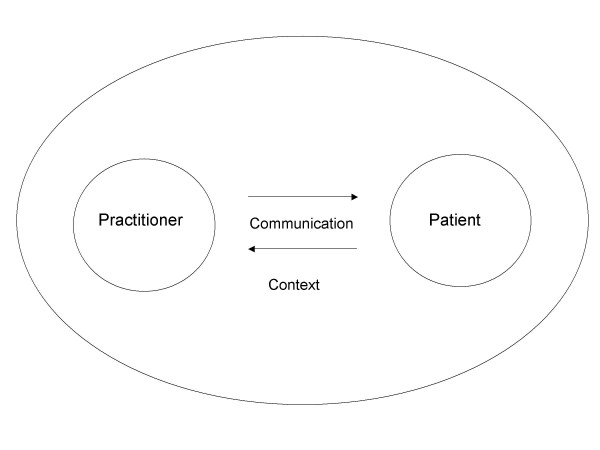
The four elements of the doctor-patient-relationship.

Our purpose was to reflect the first impression of the students on the primary health care culture. In addition, the authors analysed the doctor-patient interaction against the contextual framework (Figure 1). During the process, we quantified and analysed the presence of concepts within texts putting more emphasis on qualitative description of the contents. A native English speaker with proficiency in Finnish checked the translated citations.

The reports of 2003 and 2004 were used as reference material to check whether the findings are in line with those of 2003 reports.

## Results

The primary health care centre visit was reported as very useful or useful by 90% of all reporting students. No student reported the visit as useless or waste of time.

### Attitudes of the practitioners towards their patients

The GPs were described as: *good listener, actively present, interested, funny, cheerful, respectful, genial, warm, cosy, caring, friendly, empathic, neutral, objective, correct, effective, competent*, and *diplomatic*. Some students reported that the consultation was pleasant, even though negative expressions were used for the practitioner such as *reserved *or *distant*. Only a few students observed attitude problems, such as *a dismissive attitude, unwillingness, sneering at the patient *or *anxiety*.

Two thirds (70%) of the students reported positive or very positive attitude of GPs towards the concerns of their patients. Students reported that the GPs treated patients as human beings, not only as patients or clients. Moreover, students observed that GPs allowed patients to express themselves freely: *In my opinion doctor Y's attitude towards the patients was objective but human. She did not remain distant. When I observed her work, I felt that she really cares, and concentrates thoroughly on each and every case. She had a good contact with her patients, and the patients described their complaints to her openly. Doctor Y spared enough time for explaining facts to patients, and the patients actively inquired for more information, if anything was left unclear (43/F)*.

Students perceived that patients could feel safe and comfortable with GPs with differing attitudes: *The younger doctor was more spontaneous and quicker in a way. He normally made jokes and chatted, and at times he could act very efficiently and solve a problem quickly. Perhaps he could see who needed support, and who only needed treatment. The older doctor was very correct; his social distance to patients seemed greater, but most probably this did not adversely affect the encounter. Although he was warm to the patient, he made sure not to step on the patient's toes. This appeared as slow movements and considerateness. On the other hand this made me think the doctor was listening and allowing enough time for the patient (60/M)*.

### Doctor-patient interaction

GPs adjusted their communication at the patient's level. Medical students observed different ways of communication depending on the patient: *The doctor knows how to handle patients and is able to adapt her communication. She normally listens to a talkative patient and at times makes focusing questions. She encourages a shy patient to talk about his/her symptoms and asks more questions (55/F)*.

In some cases, GPs seemed to clarify their messages with drawings: *Doctor S gives clear and comprehensible instructions which she also reinforces with various drawings if necessary. At the end of the session she also checks with some questions that the patient has understood the message (114/F)*. But in some cases they did not give adequate guidance in health matters: *It was strange how passively patients made use of immunization services, even though they are free of charge for certain high-risk groups, and how little people know about vaccinations and other procedures. A young man belonging to the high-risk group because of his asthma came for a specific reason. He asked if he should get an influenza vaccination, and the doctor recommended it. The man suspected that such a vaccine could weaken his own resistance to diseases. Is there a principle in Finland that only the doctor knows what to do with the patient? As far as I am concerned, I want to rectify this problem. In my opinion the patient should not even need to ask what happens, information should be given automatically, but of course hectic work makes interaction difficult (122/M)*.

Several students reported GPs as having strong opinions about unhealthy life-styles, but at the same time behaving diplomatically: *The doctor was very empathic, although strict with some patients when necessary. A couple of times I noticed that the doctor looked amused when the patient asked a medically funny question. This episode did not have any importance anyway, and at the end of the day the doctor explained the medical conception patiently, and at times looked for additional information for the patient. The doctor tried to explain things clearly, and patients had the opportunity to inquire about their concerns. The doctor as a family practitioner knows the patients and thus is able to adjust his behaviour and communication style according to the patient (66/F)*.

Some students reported that the GP whose communication with ordinary patients was emphatic turned to more distant with children, mentally retarded, elderly, and socially marginalised patients. Similar aloofness was observed with accident and emergency cases.

Students reported that the working pace of GPs in accident and emergency departments is hectic: *Both doctors behaved differently in the A&E department than in the non-acute clinical sessions. In my opinion, this was merely due to the high number of patients in the A&E department, where the hectic working pace simply could not be handled. The fairly cold attitude put me off in the beginning, but afterwards I realized that it is the only way to cope with the chaotic situation (94/M)*. In addition, students perceived and reported GPs working style as mechanical and distant: *Also on the ward the same attitude of help and support was present, but one could not help getting the impression that chronic patients are like things, plants or animals, features which the doctor investigates with the nurse and introduces to a guest (89/M)*.

The computer screen and the concentration of GPs on other matters (incoming telephone calls, and other staff members entering the consultation room) were experienced as disturbing factors in doctor-patient communication: *I did not feel comfortable with the habit of all the doctors of staring at the computer screen while listening to the patient. Many times the patients, while they were still speaking, started to look at me as if they were explaining their concerns to me (63/F)*.

Students reported continuity of care as an important element of the doctor-patient-relationship: ... *Patients seem to be very open with regard to their illnesses. Doctors behave like patients' family members. Based on our discussions with patients, they feel comfortable in seeking medical help from their own family doctors and are bold enough to ask them for help, unlike in the case of an unknown doctor. Also doctors gave positive feedback about the family doctor system; it often helps when one knows the concerns of the patient in advance. So, no time is wasted on getting to know each other (54/F)*.

The reports suggest that the primary health care centre visit may have a positive effect on the early professional growth of medical students and seems to turn negative attitudes towards primary health care into more positive attitudes. Several students observed identity forming rituals; they appreciated that they were introduced to the clients as GPs' "colleagues". Similarly, wearing white uniform, stethoscopes, getting small gifts from pharmaceutical representatives etc. and recognition by other staff members were reported to help them adapt to the primary health care centre work as team members:*It was nice to dress in the white uniform for the first time and feel what it is like to be a practising doctor (114). The best thing was that I had a chance to examine sinuses or auscultate lungs and heart on my own (57)*.

## Discussion

The reports reflect perceptions of first year medical students during the 2-day primary health care centre visit. They entered the institutions with their eyes open, like newcomers in a foreign culture. Thus their observations represent a mixture of lay and professional conceptions, although the students had already gone through the introductory theoretical studies and practical exercises in communication skills prior to the primary health care centre visit.

In our study, in spite of the fact that each researcher analysed one-third of the material independently, the researchers' final classification of the material was similar, and the conclusions were parallel. No conflicting facts were found with the reference data of 2003 and 2004.

There are two main reliability problems in our study. Firstly, the students may have acquired a slightly more positive impression of the doctor-patient interaction than is actually the case, because the presence of a student in the consultation room may have made the GPs more polite and careful. Secondly, due to the timing of the health care centre visit in the first weeks of medical studies, the general practice culture may have been so fascinating and exciting to the students that they may have picked more positive than negative experiences ("honeymoon effect ").

The students' reports are well in line with previous findings that Finnish primary health care centre doctors are highly committed to their work and the concerns of their patients, but are also under heavy psychological stress caused by the ever-increasing demands made on them by the public health care system [12,14]. Medical students observed with admiration the diversity of GP work, and seemed to realise the great variety of skills and capabilities that are required in general practice.

The students regarded the two-day primary health care centre visit as useful and motivating. They reported the interaction between the GP and the patient as keys to a successful clinical encounter. They reported primary health care physicians as adaptable and skilful medical experts, but also as good supporters, even friends in some cases. The approach of the individual GPs differed, but very few were reported to have big difficulties in inter-personal relationships.

The students perceived some weak areas in the doctor-patient interaction, such as the behaviour of GPs in a busy working environment, with socially marginalized groups, and with seriously ill and dying patients.

The students seemed to have – a priori – an impression what a good GP is like, and how patients should be treated. Evidently, the early pre-clinical contact of medical students with the real general practice helps students adopt a holistic approach in their future clinical work. As a result, *they treat patients like human beings or real friends, and not like numbers, machines or strangers *[15].

## Conclusion

Our findings are in line with the previously documented importance of pre-clinical primary care orientation programme. We discovered that the positive role model given by senior general practitioners obviously strengthens the confidence of first year medical students in GP-work; with the result that working as a GP can be one of their realistic career options. We welcome more European research on the influence of the early pre-clinical family practice exposure on medical students' primary care specialty choice.

## Competing interests

The author(s) declare that they have no competing interests.

## Authors' contributions

The reports of 2002 were divided randomly into three parts of equal number. Each author (JM, PM, TV) read through his/her share. Consequently, series of working sessions took place, and consensus of the findings was achieved. JM read through the reports of 2003 and 2004 for reference, and drafted the manuscript. PM and TV contributed with their additional comments. Finally, all authors read and approved the manuscript. The reviewers' reports were discussed intensively. After consulting some additional literature, revisions were made as a joint venture.

## Pre-publication history

The pre-publication history for this paper can be accessed here:


